# Homozygous KIR2DL3 confers susceptibility to tuberculosis infection in HIV seropositive individuals

**DOI:** 10.1186/1471-2334-14-S3-P67

**Published:** 2014-05-27

**Authors:** Stalinraja Maruthamuthu, Suresh Madasamy, Jayalakshmi Mariakuttikan

**Affiliations:** 1 Department of Immunology, School of Biological Sciences, Madurai Kamaraj University, Madurai- 625021, India; 2 ART Centre, Govt. Theni Medical College and Hospital, Theni, India

## Background

Tuberculosis (TB) remains an important cause of death among patients infected with HIV. Genetic variation influences differential development of TB particularly in Immunosupressed patients. Natural Killer cells play an important role in the innate immune system by providing the first line of defence against viral infections and tumors. Their activity is partially controlled by distinct inhibitory and activating killer Ig like receptors (KIR) that recognize specific ligands on potential target cells. Objective of this study is to determine the association between KIR and HIV co-infection.

## Methods

This nested case control study comprised of 68 HIV+ individuals (34 treatment naive HIV-1 slow progressors and 34 on ART patients with TB co-infection). Genomic DNA was extracted from peripheral blood samples by salting out procedure and KIR genotyping was performed for 15 KIR genes (Activating and Inhibitory) by Multiplex PCR sequence specific primer method.

## Results

All 15 KIR genes were observed in this study cohort. We could observe more number (>4 aKIR) of activating KIR genes in HIV+ TB– individuals (88%) when compared with HIV+ TB+ patients (74%). Homozygosity for KIR2DL3 was more frequent in HIV+ TB+ patients (64%) than HIV+ TB– individuals (32%) which may confer susceptibility to co-infection.

## Conclusion

NK cell receptor KIRs limits HIV disease progression. In the present study, HIV positive individuals who were homozygous for inhibitory KIR2DL3 are at higher risk of acquiring TB co-infection and disease progression with decreased CD4 cells.

